# Drought-Induced Root Pressure in *Sorghum bicolor*

**DOI:** 10.3389/fpls.2021.571072

**Published:** 2021-02-03

**Authors:** Sarah Tepler Drobnitch, Louise H. Comas, Nora Flynn, Jorge Ibarra Caballero, Ryan W. Barton, Joshua Wenz, Taylor Person, Julie Bushey, Courtney E. Jahn, Sean M. Gleason

**Affiliations:** ^1^Department of Soil and Crop Sciences, Colorado State University, Fort Collins, CO, United States; ^2^Water Management Research Unit, Agricultural Research Service, USDA, Ft. Collins, CO, United States; ^3^Department of Agricultural Biology, Colorado State University, Fort Collins, CO, United States

**Keywords:** water relations, xylem transport, root pressure, RNA-Seq, agriculture, transporter, aquaporin

## Abstract

Root pressure, also manifested as profusive sap flowing from cut stems, is a phenomenon in some species that has perplexed biologists for much of the last century. It is associated with increased crop production under drought, but its function and regulation remain largely unknown. In this study, we investigated the initiation, mechanisms, and possible adaptive function of root pressure in six genotypes of *Sorghum bicolor* during a drought experiment in the greenhouse. We observed that root pressure was induced in plants exposed to drought followed by re-watering but possibly inhibited by 100% re-watering in some genotypes. We found that root pressure in drought stressed and re-watered plants was associated with greater ratio of fine: coarse root length and shoot biomass production, indicating a possible role of root allocation in creating root pressure and adaptive benefit of root pressure for shoot biomass production. Using RNA-Seq, we identified gene transcripts that were up- and down-regulated in plants with root pressure expression, focusing on genes for aquaporins, membrane transporters, and ATPases that could regulate inter- and intra-cellular transport of water and ions to generate positive xylem pressure in root tissue.

## Introduction

Root pressure is a phenomenon that has long puzzled plant scientists (Hales, 1727; Priestley, 1920; Pickard, 2003; Wegner, 2014; Singh, 2016). It is observed in the xylem of a paraphyletic array of woody and herbaceous dicots and monocots, from *Acer* sp. to kiwifruit to *Zea mays* (O'Leary, 1965; Fisher et al., 1997; Enns et al., 2000; Ewers et al., 2001; Clearwater et al., 2007). It is difficult to measure *in vivo*, particularly for herbaceous plants but with high variability among species, genotypes within species and temporally, is intriguing to better understand (Grossenbacher, 1938; Fisher et al., 1997; McCully, 1999; Ewers et al., 2001; Sperry et al., 2008; Gleason et al., 2017; Comas et al., under review). Despite numerous observations and manipulative experiments, the molecular mechanism(s) generating positive pressure in a xylem hydraulic system dominated by negative tension remain unknown (Fiscus and Kramer, 1975; Schenk et al., 2021). Among several hypotheses, one posits that root pressure is generated by xylem parenchyma acting as an osmometer, structuring the osmotic potential of cell compartments to passively pull water into the mature xylem from the apoplast or bulk medium (Enns et al., 2000). Another suggests a hydraulic pressure scenario driving water into vessels via membrane asymmetry and compartmentalization of forces (Pickard, 2003). Yet another suggests a “futile cycling” system in which water molecules are repeatedly scavenged from the apoplast back into the symplastic xylem parenchyma and pumped into the mature xylem, requiring energy expense (Pickard, 2003; Wegner, 2014). All of these mechanisms are in stark opposition to the mechanism of xylem water flow during transpiration, in which water is drawn through the xylem apoplast along a hydrostatic pressure gradient (Steudle, 2000). Importantly, generation of root pressure also must involve a mechanism for preventing leakage of water out of the xylem into the bulk medium (soil) during times of positive pressure. This process may be a combination of regulation across mechanical barriers (e.g. the Casparian strip) or the abovementioned “futile cycling” in which water is scavenged from the apoplast back into the symplast (Lee et al., 2004; Wegner, 2014).

There is also considerable debate as to whether root pressure is an evolutionary positive or neutral trait. A potential positive adaptive function of root pressure may be to refill xylem embolisms in water- or salt-stressed plants under drought, and after freezing events. Indeed, embolism refilling has been correlated with root pressure in multiple systems, including ferns (Holmlund et al., 2020), poplar (Secchi and Zwieniecki, 2014), birch (Hölttä et al., 2018), grapevine (Barrios-Masias et al., 2015; Knipfer et al., 2015), and maize (Tyree et al., 2008; Gleason et al., 2017). However, embolism refilling has also been observed in the absence of root pressure (Stiller et al., 2005; Choat et al., 2015; Knipfer et al., 2016) and attributed to other mechanisms such as biomechanical capillary action (Rolland et al., 2015). Historically, most researchers have assumed that both root pressure and embolism refilling occur at night, when most plants have low or no transpiration. During this time xylem is under little to no tension, and the generation of even slight positive pressure could potentially refill embolized conduits. However, embolism refilling has also been observed in the early afternoon, easily the most stressful part of the day in *Zea mays* (McCully, 1999). Root pressure may be diurnally constitutive but simply masked during the day by the magnitude of negative hydrostatic forces.

Most work on root pressure to date has focused on establishing the presence or absence of root pressure in species of interest, with some functional studies using various inhibitors to regulate root pressure magnitude in plants subjected to osmotic stress (Heindl et al., 1982; Ranathunge et al., 2003; Wright et al., 2004). While these plant physiological studies are important, transcriptomic tools represent another approach to identifying the mechanisms of root pressure. Using RNA-seq, researchers have been able to observe differential expression of both known and unknown genes during the activation of plant functions in response to stress (Buchanan et al., 2005; Johnson et al., 2014; Reddy et al., 2015; Kadam et al., 2017). A transcriptomic approach to identify the molecular mechanisms of root pressure generation is certainly exploratory but can be focused on the regulation of genes responsible for osmotic adjustment and transmembrane transport. Of particular interest are aquaporins, a class of proteins facilitating water transport across cellular membranes that is already known to be involved in hydraulic regulation (Henzler et al., 1999; McElrone et al., 2007; Vandeleur et al., 2009; Secchi et al., 2011; Meng et al., 2016; Shi et al., 2016).

The causal dynamics of the relationship between drought stress, drought recovery and root pressure are largely unknown. If root pressure ameliorates embolism in drought stressed plants, is it initiated by drought or constitutively present in well-watered plants as well? Do plants with greater root pressure have greater stem or leaf hydraulic conductance due to reduced embolism and higher parenchymal turgor? Does greater root pressure under drought support less negative leaf water potentials and better photosynthetic function? If root pressure is generated by root tissues, do biometric root traits correlate with root pressure? Is root pressure generated by a certain root class, such as fine roots, which have a lesser degree of suberization and barriers to apoplastic water movement (Guo et al., 2008; McCormack et al., 2015)? Finally, of utmost importance—do plants or species with robust root pressure show empirically greater survivorship, growth, or yield under drought conditions than counterparts with lower or no root pressure?

To investigate the mechanism and function of root pressure, we carried out a drought experiment in the greenhouse using *Sorghum bicolor. S. bicolor* is an important drought tolerant grain traditionally cultivated in Africa and Asia, with subspecies and varieties used for silage, biofuels, and human consumption (Rosenow et al., 1983; Doggett, 1988; Hariprasanna and Patil, 2015). As rising temperatures, unpredictable precipitation regimes, and drought intensify as the result of climate change, drought-tolerant *S. bicolor* is of great interest to plant breeders (Boyer, 1982). As an important global crop, its annotated genome is readily available, aiding in transcriptomic studies (Buchanan et al., 2005; McCormick et al., 2018). Finally, a wide range of cultivars are available within *S. bicolor*, providing marked differences in habit, physiology, and drought-tolerance (Turner et al., 2016; Miller, 2018).

We grew six genotypes of *S. bicolor*, three drought tolerant and three susceptible, under well-watered conditions, and then subjected a subset of the plants to a 2-week drought period. To understand the relationship between root pressure magnitude and plant physiological traits, we destructively harvested plants after drought and re-watering treatment to measure root pressure, leaf water potential, leaf vascular embolism, and root/shoot biometric traits. Finally, we explored molecular mechanisms of root pressure by sampling the root transcriptome of drought stressed and control plants immediately preceding root pressure measurement. Using differential expression analysis, we searched for genes that could be involved in inter- and intra-cellular transport of water and ions to generate positive xylem pressure in root tissue.

## Methods

### Location and Dates of Study

The study was carried out in the greenhouse at the USDA Crops Research Laboratory in Fort Collins, CO. Germinated seedlings were planted into experimental pots on January 30, 2018, and destructively harvested during the week of April 1, when most plants had 5-6 fully expanded leaves.

### Selection of *S. bicolor* Varieties

Six diploid lines of grain *S. bicolor* (bred for grain yield) were selected to span a broad range of variation in morphology, grain yield, and drought tolerance (Rosenow et al., 1983; Dahlberg, 2000; Evans et al., 2013; Miller, 2018). Rtx430 (Feterita x Zerazera: Sudan/Ethiopia), BTx623 (Kafir: Southern Africa), and Tx7000 (Durra: Sudan/Ethiopia/Egypt) are pre-flowering drought tolerant; BTx642 (Caudatum: North-central Africa), IS3620C (Guinea: West Africa), and SC56 (Caudatum) are pre-flowering drought susceptible. Susceptibility/tolerance are inferred from the effect of pre-flowering drought on final grain yield. RTx430, BTx623, and Tx7000 have the highest water use efficiency (WUE), followed by BTx642 and IS3620C, with SC56 having the lowest WUE (Turner et al., 2016).

### Seed Germination

Seeds of the six genotypes were germinated (25°C, dark) in Petri dishes on filter paper with fungicide solution (Maxim XL, Syngenta) for 1 week prior to transplanting to pots. When seedling shoots and roots were each 2 cm long, they were transplanted into 7.57 L black polypropylene pots filled with fritted clay (Profile® Greens Grade^™^ in Emerald, PROFILE Products, LLC., Buffalo Grove, IL, USA). Fritted clay is a non-nutritive substrate with excellent water retention that detaches easily from plant material when dried, making it ideal for studies of root biomass and morphology.

### Experimental Setup

**Figure 1:**
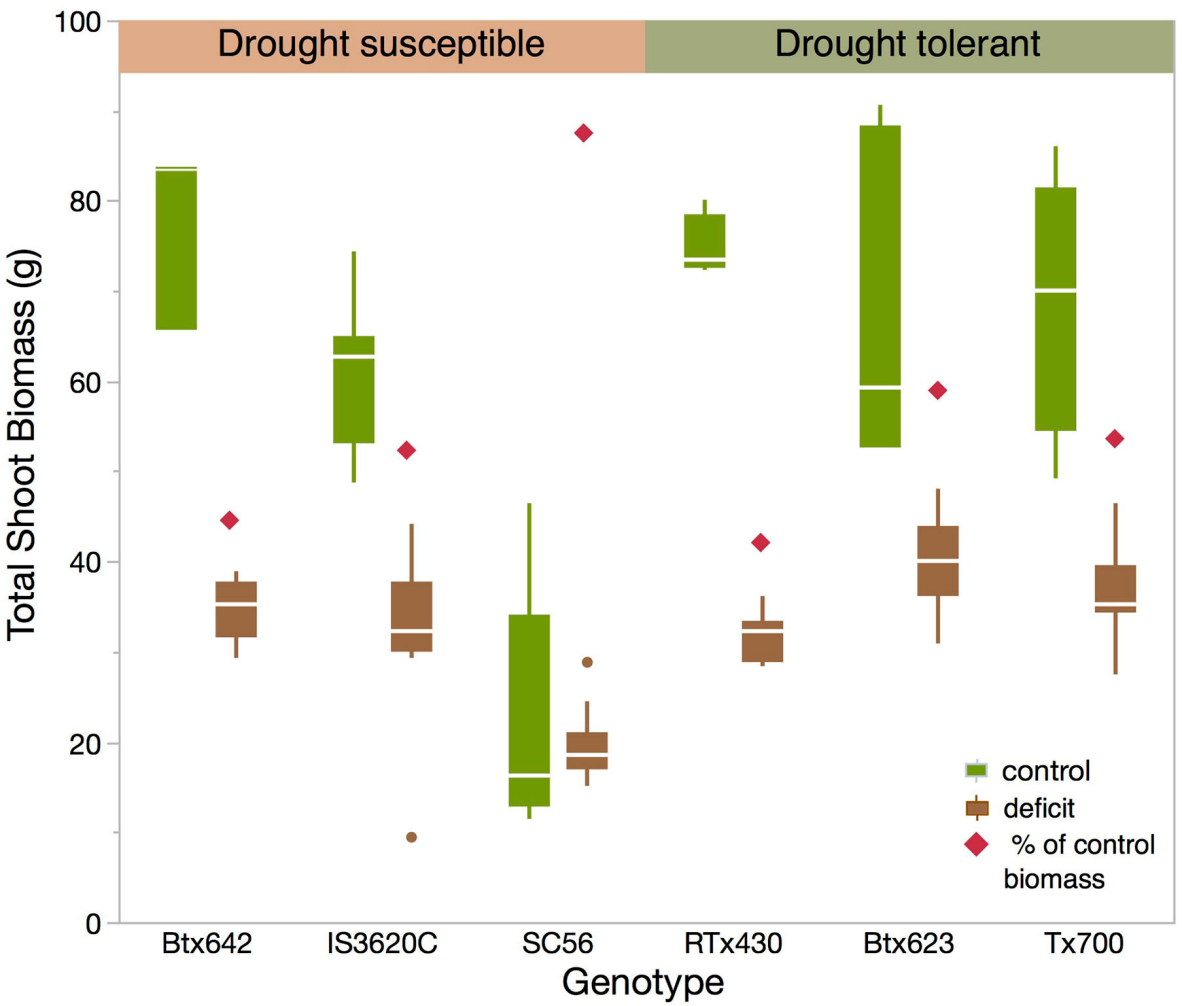
Boxplot of total shoot biomass achieved before harvest by each genotype grown either under control or deficit conditions, as well as percent deficit shoot biomass for each genotype.

The 7.57 L pots were arranged into two rows with nine groups of six pots with randomized placement of each genotype within each group (Supplementary Figure 1). Two groups of four pots were added to both ends of the bench to provide buffer. Four groups of the genotypes ended up missing two genotypes when seedlings failed after transplanting and thus were lost replicates. These plants were replaced with extra seedlings of an available genotype and treated as an additional buffer plant (Figure 1). Pots were irrigated by polypropylene irrigation lines that delivered fertigated water via an inline Dosatron (model D14, Dosatron Inc., Clearwater, FL) to 3.8 L/min emitters (one emitter per pot). A closed 227 L drum of concentrated fertilized irrigation water was prepared using municipal water, Gro-Mor fertilizer (Gro-Mor Plant Food Co., Inc., Leola, PA), and CaNO_3_ each month to supply the Dosatron. After the initiation of the deficit treatment, control blocks were watered by hand.

### Initial Growth Conditions

Experimental plants were established and grown in well-watered, nutrient replete conditions for 8 weeks (Supplementary Figure 2). Pots were fertigated with 80 ppm nitrogen (N) solution to holding capacity each afternoon at 1,600 h. Plants were given a 14/10 h day/night regime using banks of LED lights emitting ~300 μmol photons/m^2^/s. During natural daytime, sunlight increased ambient irradiance to a maximum of 1,000 μmol photons/m^2^/s. The greenhouse air temperature was maintained at 28°C (day) and 17°C (night), and relative humidity was observed to be between 40 and 50% during the day and 60–70% at night.

### Growth Conditions During Deficit Treatments

After 8 weeks of growth at 100% replenishment of daily evapotranspiration (ET), water deficit treatments were established on March 19, 2018 (Supplementary Figure 2). Five blocks were established as controls, continuing to receive 100% ET and 80 ppm N via hand watering (Control). Control plants were weighed daily to track mean ET as it increased with plant size and for calculations of deficit watering amounts. The remaining 8 blocks were watered at 30% of control ET and 80 ppm N (Deficit). These experimental treatments were implemented for 2 weeks, after which plants were destructively measured.

### Non-destructive Measurements During Deficit Treatment

Non-destructive plant biometrics (leaf length, number of leaves, tiller length, and tiller count) were measured at the start of deficit treatments (after 8 weeks of growth) and at final harvest (after 10 weeks of growth).

Gas exchange measurements were taken during week 9 of the experiment from the following subset of treatments: 3 control blocks, 4 deficit + high N blocks, and 4 deficit + low N blocks. Measurements were made between 0800 and 1,100 h, the period when leaves were most photosynthetically active. From each plant, the 3rd collared leaf from the top was placed into a gas exchange cuvette (LI-6400–40, LI-COR Biosciences, Lincoln, Nebraska, USA), avoiding the midrib. Leaves were permitted to acclimate to conditions in the cuvette (1,200 PAR, 25°C T_leaf_, 400 ppm CO_2_, and 70% RH) for 10 min or more until the rate of photosynthesis remained constant, at which point an instantaneous measurement of maximal photosynthetic rate (A_max_) was made. Temperature and relative humidity were kept between 24.6–30.2°C and 45–86%, respectively, for all measurements.

### Re-watering Treatment and Assessment of Hydraulic Traits

Following the 2-week drought period, a subset of 72 plants was re-watered to 100% ET (19 control plants, 16 deficit plants), 50% ET (17 deficit plants), or kept dry (six control plants, six deficit plants). All 72 plants were then individually wrapped in 1 × 1.5 m bags made of reflective Mylar plastic, which excluded all light and retained all moisture, to down-regulate photosynthesis and close stomata (Holbrook, 2001). The internal pressure of the plants was allowed to equilibrate to a steady state for 1 h while covered by Mylar.

### Leaf Water Potential Determination

After the 1 h equilibration under Mylar, the *Ψ*_L_ of each plant was determined. One upper leaf (generally the 2nd collared leaf from the top) was cut from the plant, wrapped with a damp cloth, trimmed along the midrib so the midrib could be inserted through the chamber lid and transferred immediately to a Scholander pressure chamber (Model 3005, Soil Moisture Equipment Corp, Santa Barbara, CA, USA) to determine *Ψ*_L_.

### Leaf Embolism Determination

Simultaneously another leaf (generally the first collared leaf from the top) was sampled to measure incidence of leaf embolisms. The leaf was cut with a sharp pair of scissors. Adhesive was immediately applied to the cut surfaces (Loctite Liquid, Henkel, Corp., Westlake, OH, USA) to reduce air entry. Leaves were placed in sealed plastic bags and kept in a dark insulated box until *Ψ*_L_ could be measured, generally within 1 h of collection. Bagged leaves were taken to the x-ray micro-computed tomography (μCT) facility (The CSU Flint Animal Cancer Center, Colorado State University, Fort Collins, CO, USA) and scanned at ~5-μm pixel^−1^ resolution through the leaf tissue and midribs (VivaCT 80, Scanco Medical AG, Bruttisellen, Switzerland). Leaves were bundled together and scanned in one run.

Embolized vessels were clearly identifiable in the images, whereas water-filled vessels were indistinguishable from the hydrated tissues surrounding them. Sample μCT images are provided in the Supplementary Figure 3. The total number of meta- xylem vessels in each scanned leaf was determined from stained (0.01% safranin-o, Fisher Scientific, Nazareth, NJ, USA) and photographed sections taken on the same leaf sections using a Nikon SMZ-U dissecting microscope (Nikon, Tokyo, Japan). The fraction of total conductance lost was assumed equal to the number of gas-filled meta-xylem vessels (from μCT), relative to the total number of meta-xylem vessels (from photographs).

### Root Tissue Sampling for Transcriptomic Analysis

Finally, after leaf sampling, fine roots were sampled for transcriptomic analysis. An hour after re-watering, a 10 × 5 cm window was cut into the bottom third of the polypropylene pot and a large clump of fine roots extracted with sterile forceps. Fine roots were quickly rinsed to remove soil and mineral precipitates, blotted dry with a sterile Kimwipe (Kimberley-Clark Corporation, Irving, TX), and chopped roughly into 0.5 cm lengths with a sterile razor blade. Chopped roots were immediately transferred to 10 mL vials containing RNALater stabilization solution (Invitrogen, Carlsbad, CA), refrigerated for 24 h, and subsequently frozen at −80°C until extraction and sequencing. A subset of roots was reserved and frozen for assessment of root viability in dry vials.

### Root Pressure Measurements

Directly following internal plant pressure equilibration and leaf/root sampling, plants were moved to a dark room, uncovered, and the shoot removed 3 cm above the brace roots. Immediately after each shoot was removed from the plant, a pre-weighed cotton pad was placed on the cut surface of the stem to absorb root flow (sap driven by root pressure). Cotton pads were removed after 10 min and re-weighed to calculate the quantity of sap pushed out of the root system through the cut stem (“root flow rate”) (Zhang et al., 1995). Directly after measuring root flow rate, the decapitated shoot base was capped with nested sections (~2 cm) of polypropylene tubing and sealed with Loctite adhesive. The cap terminated in a 0–0.21 MPa pressure transducer (model PX26-030DV, Omega Engineering, Inc., Stamford, CT, USA) (Sperry, 1983). Root pressure measured by this transducer was then recorded for 24–48 h in total darkness via a data-logger (CR1000, Campbell Scientific, Inc., Logan, UT, USA) and the maximum pressure documented during this time was used for analyses. We note that our measured root flow rates likely overestimate the flow rates that would occur in entire intact plants because they do not include resistances associated with xylem conduits as well as extra-xylary resistances in stems and leaves.

### Plant Biomass Measurement

After all destructive measurements were performed, intact roots were removed from pots, thoroughly rinsed and collected through a 2 mm sieve to remove all soil particles. Leaves, with the exception of the leaf section used for microCT, were scanned with a LI-3100C leaf area meter (LI-COR Biosciences, Lincoln, Nebraska, USA). All plant material was dried at 60°C and weighed. Root systems were then rehydrated for 24 h and separated into coarse (> 0.15 mm in diameter) and fine roots, a subset of which was scanned and analyzed in WinRHIZO (Regent Instruments, Inc., Canada), dried and reweighted to quantify specific and total root length of each class.

### Transcriptomic Analysis of Root Tissue

After root pressure data was analyzed, samples from two genotypes, both watering treatments, and three ranges of root pressure magnitude were chosen to be processed for transcriptomic analysis. Three groups were established to carry out differential expression analysis: Control, Low (consistently watered control plants with root pressure expression < 5.0 KPa), Deficit, Low (deficit-grown plants with root pressure expression < 5.0 KPa), and Deficit, Mid (deficit-grown plants with root pressure expression between 5.0 and 10.0 KPa). Three individual plant samples were sequenced for each treatment except Control, Low, for which we only took two samples.

RNA from root tissue was extracted and sequenced by Amaryllis Nucleics (Oakland, CA) using the protocol of Townsley et al. (2015). RNA-Seq libraries were prepared using a high-throughput Illumina RNA-Seq library extraction protocol (Kumar et al., 2012). The enriched libraries were then quantified on an Analyst Plate Reader (LJL Biosystems) using SYBR Green I reagent (Invitrogen). Once the concentration of libraries was determined, a single pool of all the RNA-Seq libraries within each block was made. The pooled libraries were run on a Bioanalyzer (Agilent, Santa Clara) to determine the average product size for each pool. Each pool was adjusted to a final concentration of 20 nM and sequenced on a single lane on an Illumina Hi-Seq 2000 flow cell as 50 bp single-end reads. Any failed samples from the five blocks were run on two additional lanes.

### Analysis

Statistical analysis of plant trait responses to imposed treatments was carried out in JMP 13 (SAS Institute Inc., Cary, North Carolina USA) and in R. The dataset was split into subsets (see Table 1 notes) to evaluate specific hypotheses given the available sample sizes. Distributions of trait residuals were assessed for non-normality and transformed as needed. Traits for each ANOVA were examined for unequal variances within each treatment factor combination and the validity of differences between means with unequal variances were checked using Welch's ANOVA and, when necessary, non-parametric pair-wise comparisons using Wilcoxon's method. In all cases, non-parametric tests supported the results of initial ANOVA analyses; thus, for simplicity, the ANOVA results are reported. Finally, to visualize which traits strongly covary with root pressure, we performed a Principal Component Analysis (PCA) in R using the prcomp() function. We chose the following seven variables for the PCA based on cluster analysis (Proc VARCLUS, SAS Institute Inc., Cary, North Carolina USA) and a priori hypotheses of which traits might contribute to different drought-adaptive strategies: root pressure, green shoot biomass, number of tillers, mean tiller height, leaf water potential, ratio of fine: coarse root length and root mass fraction. Mean tiller height is a proxy for total plant height, with which it was strongly correlated in the cluster analysis.

**Table 1:** ANOVA of total biomass, leaf water potential, and root pressure magnitude by genotype, drought treatment, re-watering treatment, major root type, and interactions.

**Response variable**	**Explanatory variable**	**Nparm**	**DF**	**Sum of Squares**	**F Ratio**	**Prob > F**	**Dataset notes**
Total biomass	GENOTYPE	5	5	39436.089	30.9788	<0.0001	
Total biomass	H20_TREAT	1	1	17746.743	69.7043	<0.0001	
Total biomass	GENOTYPE^*^H20_TREAT	5	5	5796.321	4.5533	0.0009	
Leaf water potential	GENOTYPE	5	5	22.167734	1.8494	0.1206	Re-watered plants only
Leaf water potential	H20_TREAT	1	1	7.786381	3.248	0.0777	Re-watered plants only
Leaf water potential	H20_TREAT^*^GENOTYPE	5	5	7.132211	0.595	0.7038	Re-watered plants only
sqrt(Root pressure)	H20_TREAT	1	1	11.631048	3.6098	0.0657	100% and 0% rewatering levels only
sqrt(Root pressure)	REWATER(H20_TREAT)	1	1	66.425874	10.3079	0.0003	100% and 0% rewatering levels only
Root pressure (KPa)	REWATER	1	1	959.3009	6.983	0.0152	Deficit plants only
Root pressure (KPa)	GENOTYPE	5	5	1528.3695	2.2251	0.0898	Deficit plants only
Root pressure (KPa)	GENOTYPE^*^REWATER	5	5	4053.8495	5.9018	0.0015	Deficit plants only

Preprocessing of the Illumina RNASeq raw reads was done using fastp (Chen et al., 2018). Quality of the reads before and after the fastp processing was assessed using fastqc (Andrews, 2010). Reads were mapped to the *S. bicolor* genome (v.3.1.1) from the Joint Genome Institute (McCormick et al., 2018). Assembly of the transcriptome was performed using the Tuxedo Protocol (Trapnell et al., 2012). The included versions of Bowtie, TopHat and Cufflinks were 2-2.3.4.2, 2.1.1, and 2.2.1, respectively. Default parameters were used, with the exception of the following: min-intron-length 4 and max-intron-length 1,900. Intron length was chosen based on Panahi et al. (2014). Functional annotation was obtained for a subset of the transcriptome by BLASTing annotated *S. bicolor* genes to all members of the Poaceae in the NCBI Gene Database. To assess the similarity of transcriptomes within treatments, and thus the validity of choosing those treatments for differential expression analysis, we performed a PCA in the DESeq package in R on raw counts (Supplementary Figure 4). Based on this PCA, 2 outliers were removed (one each from the Deficit, Low, and Deficit, Mid treatments). Differential analysis was then performed using DESeq on the remaining six samples (two in each treatment).

We observed significant differential expression between the three treatments and subsequently searched this subset of genes for functions with possible relevance to the creation of and maintenance of elevated root pressure. We searched our functional annotation for aquaporins, membrane-bound transporters in the plasma or vacuolar membrane, enzymes involved in carbohydrate metabolism, osmotic regulation, or vascular transport, and ATPases (necessary for aquaporin gating). We further carried out a Gene Ontology (GO) enrichment analysis using topGO in R on our annotated subset of genes to illuminate broader physiological trends differentiating the three treatments.

## Results

### Genotype Response to Deficit Conditions

The six *S. bicolor* genotypes demonstrated distinct morphologies and growth potential in both control (well-watered) and drought treatments. Final total biomass varied significantly as a function of watering treatment and genotype with significant interaction between these effects (ANOVA, Table 1). All five genotypes other than SC56 produced significantly greater biomass under control conditions (88.6–115.8 g mean dry weight) than deficit conditions but did not differ significantly among one another within the control treatment (Figure 1, Tukey's HSD). SC56 under control conditions produced a mean total biomass of 33.6 ± 7.1 g, not statistically different from its total biomass under deficit (37.8 ± 4.3 g). Under deficit conditions, Tx700 (a “drought-tolerant” genotype) produced the greatest mean total biomass (79.9 ± 4.0 g), significantly >SC56 and IS3620C (58.0 ± 4.3 g, both “drought susceptible” genotypes), but not statistically different from other genotypes of intermediate performance (BTx642 = 78.1 ± 4.8 g, “susceptible”; BTx623 = 76.2 ± 4.3 g, “tolerant”; RTx430 = 62.0 ± 5.0 g, “tolerant”).

The six genotypes did not significantly vary in *Ψ*_L_ after re-watering (Table 1). Plants that received any level of re-watering (30–100% of daily ET) recovered to a statistically similar mean *Ψ*_L_ (−0.28 to −0.42 MPa), which was significantly less negative than either control plants with zero re-watering (−0.64 ± 0.053 MPa) and deficit plants with zero re-watering (−1.45 + 0.084 MPa, nested ANOVA, re-watering level nested within deficit treatment, *p* < 0.0001).

### Induction of Root Pressure by Drought

**Figure 2:**
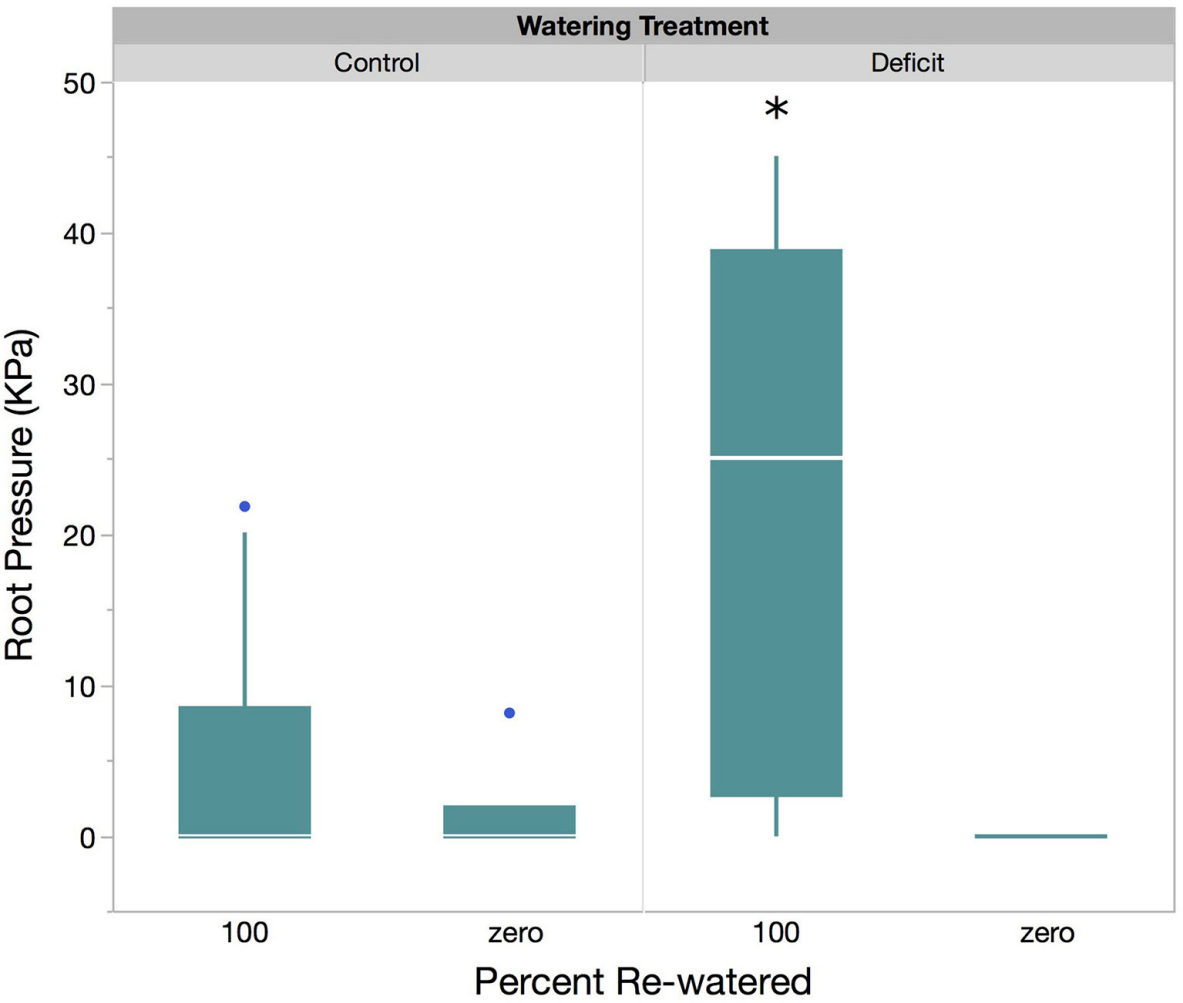
Boxplot of root pressure (KPa) measured in control plants vs. deficit plants. Directly before measurements, sample plants were re-watered with 100% of their daily evapotranspiration loss, or not re-watered at all.

As observed in previous studies, generation of root pressure depended on the presence of ambient soil water content (Kramer, 2006; Gleason et al., 2017); thus, root pressure was only observed in plants that were re-watered prior to measurement (ANOVA, Table 1, Figure 2). *S. bicolor* grown in the well-watered (control) treatment either generated no root pressure or very little (similar to pressures observed in deficit plants that were not re-watered, up to 4.72 kPa). Plants grown in the water deficit treatment generated a range of high root pressures after re-watering (mean pressure 22.25 ± 3.21 kPa, ANOVA, Table 1, Figure 2).

**Figure 3:**
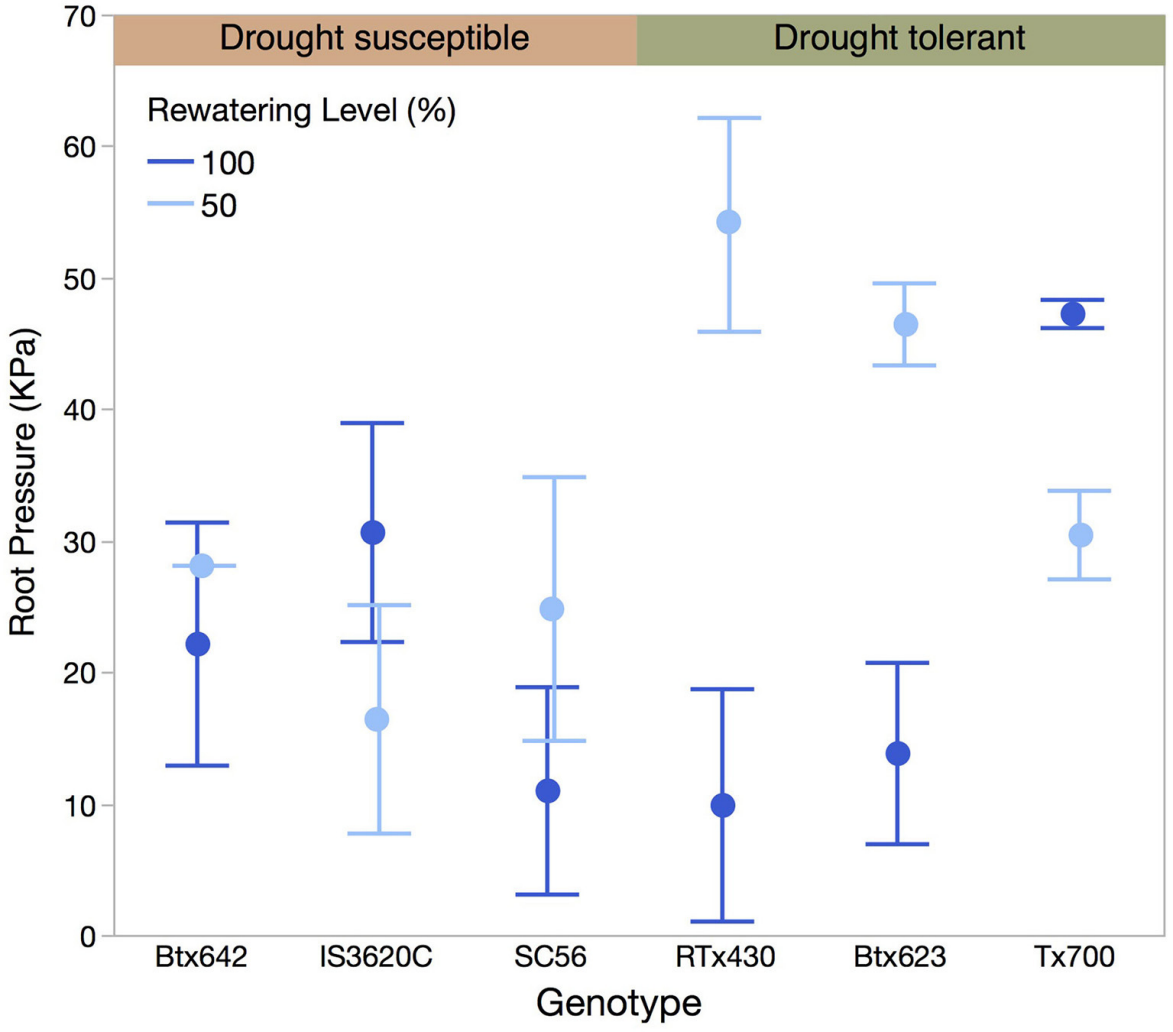
Root pressure (KPa) by genotype in samples re-watered before measurement with 50 or 100% of daily evapotranspiration loss. Error bars represent standard error.

Within the deficit treatments there was high variation in individual root pressure measurements, unexplained by either genotype or re-watering level (50 vs. 100% of daily ET, ANOVA, Table 1, Figure 3). There was, however, a significant interaction between genotypes and the degree of re-watering on root pressure, with each genotype showing a significantly different response to the degree of re-watering (ANOVA, *p* = 0.0015, Table 1, Figure 3). Two of the three drought tolerant genotypes showed lower root pressure generation under 100% re-watering than under 50% re-watering.

### Relating Root Pressure to Other Plant Traits

**Figure 4:**
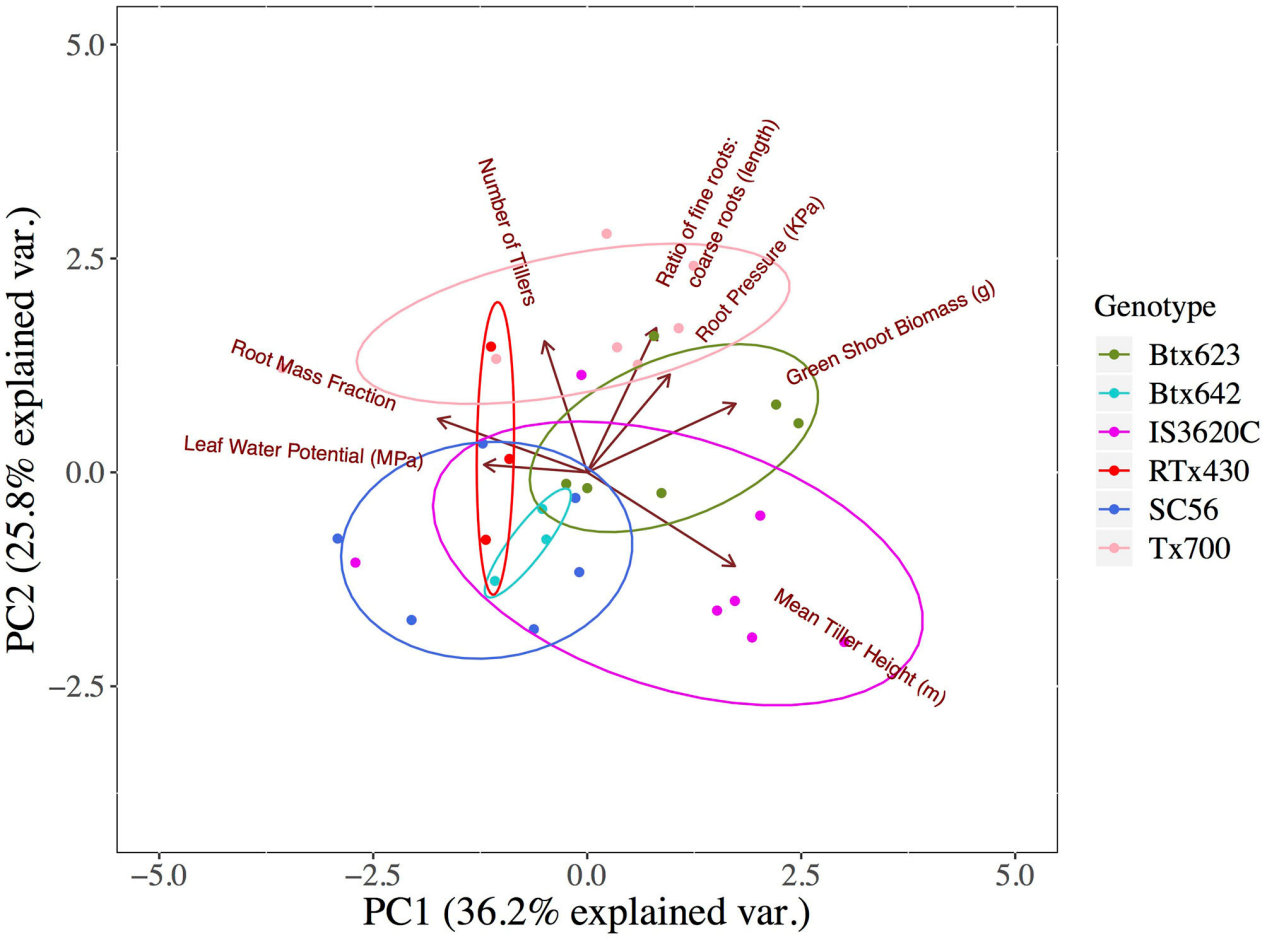
Principal component analysis of re-watered plants in the Deficit treatment. Loading arrows indicate the magnitude and direction of trait variation (7 traits) underlying the sample distribution. Ellipses indicate groupings by genotype.

Two principal components accounted for 62% of the total variation among seven traits in a PCA across deficit plants of all genotypes that had been re-watered (the group in which the root pressure was mainly observed). Among these components, there was a general association of greater root pressure, greater proportional investment in fine root length (greater ratio of fine:coarse root length), and larger green shoot biomass (Figure 4).

Among individual correlations with this same data, greater root pressure was correlated with greater green shoot biomass, total shoot biomass, and total plant biomass (Supplementary Figure 5). There was no general individual correlation between root pressure and total root biomass, coarse or fine root biomass, *Ψ*_L_, photosynthetic rate, or number of leaf emboli (Supplementary Figure 5).

Root pressure was also correlated with root flow, the measure of the overall mass of sap exuded in the first 10 min after de-topping. Root flow was correlated with total root and coarse root biomass, total leaf area, green and total shoot biomass, and total plant biomass (Supplementary Figure 5).

**Figure 5:**
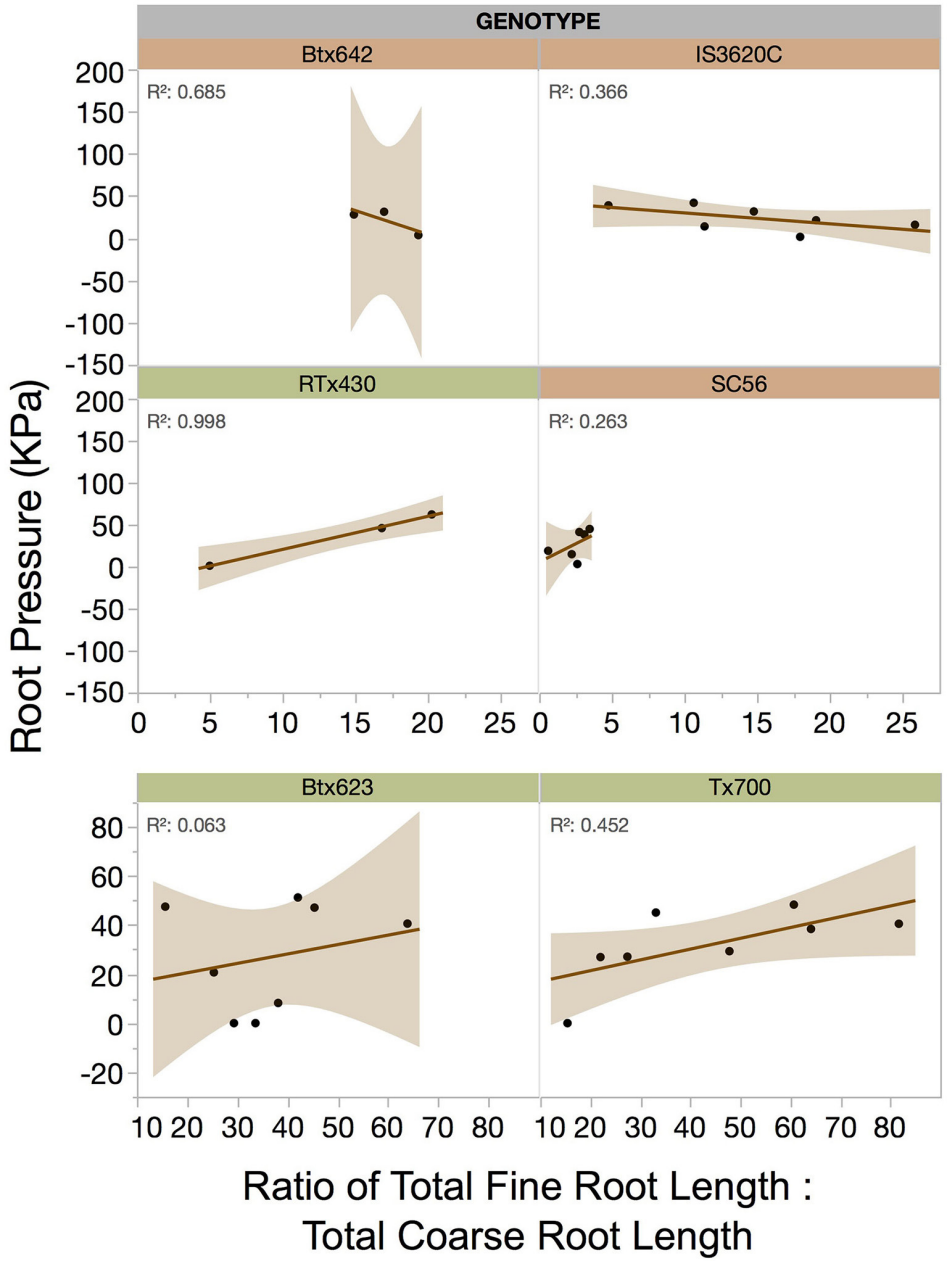
Relationship between root pressure (KPa) and the root system's relative allocation to fine roots (ratio of total fine root length to total coarse root length, unitless), by genotype. Orange genotypes (Btx642, IS3620C, and SC56) are drought susceptible, and green genotypes (RTx430, Btx623, and Tx700) are drought tolerant. Note that Btx623 and Tx700 (bottom panels) have much higher proportions of fine roots than the other genotypes. Dotted regression lines indicate non-significant relationships.

Among genotypes there were qualitatively different relationships between root pressure and the proportion of fine root investment (Figure 5). Interestingly, Btx642 and IS3620C, which are both drought-susceptible genotypes from mesic central Africa, had a slight negative relationship between root pressure and proportion of fine root investment, whereas the drought-tolerant genotypes generally displayed greater root pressure with greater fine root investment (Figure 5).

### Transcriptomic Profiling of Root Pressure Expression

**Figure 6:**
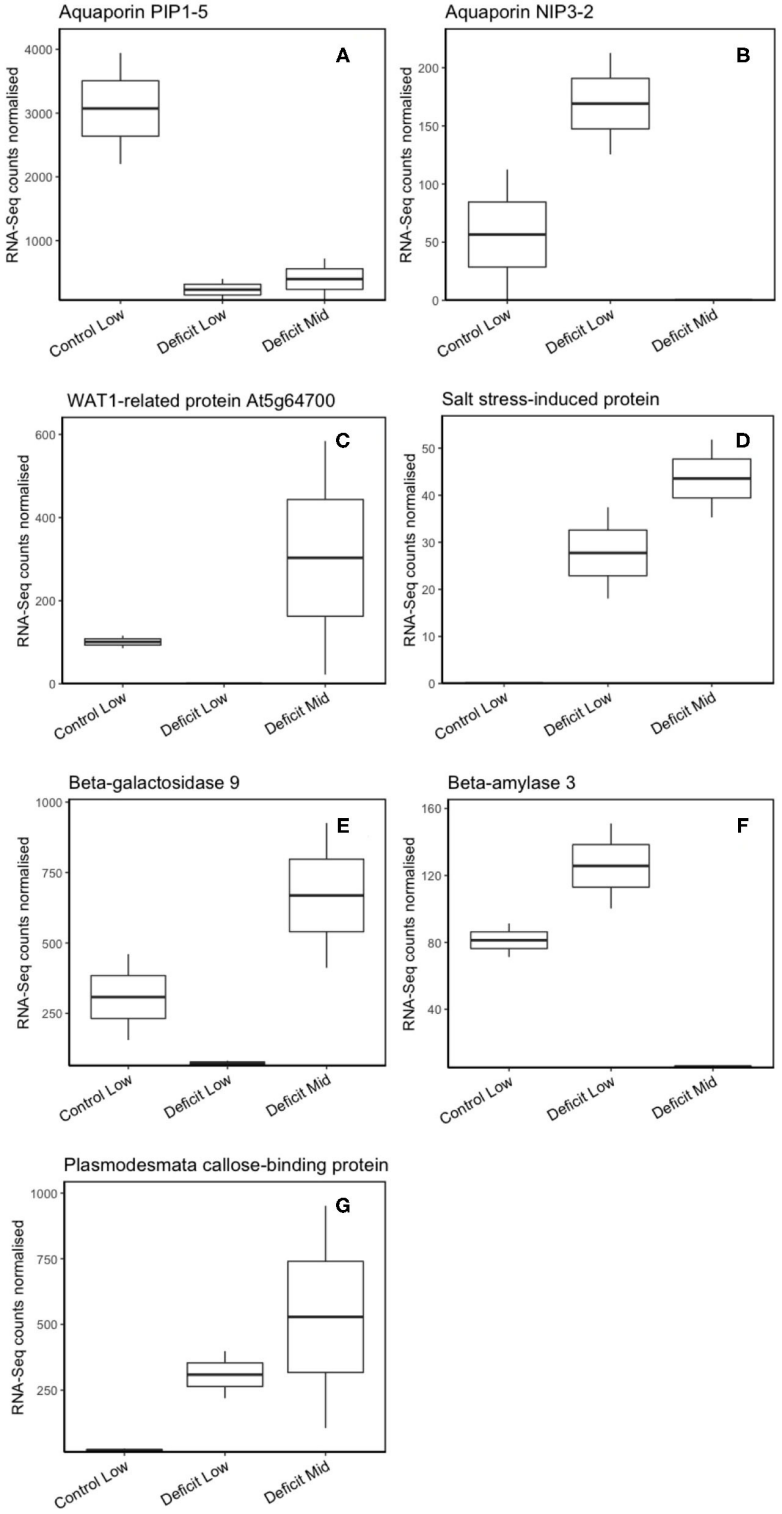
Normalized RNA-Seq counts of gene transcripts in each of three treatment groups (Control Low, Deficit Low, and Deficit Mid) for the following differentially-expressed genes: **(A)** PIP1;5, **(B)** NIP2;3, **(C)** WAT1-related protein Atg64700, **(D)** Salt stress-induced protein, **(E)** Beta-galactosidase 9, **(F)** Beta-amylase 3, **(G)** Plasmodesmata callose-binding protein.

After screening for data quality, we retained an average of 24.6 million reads per sample for use in transcriptome profiling. RNA sequencing provided a rich dataset of both unknown and annotated expressed genes. The data discussed in this publication were deposited in NCBI's Gene Expression Omnibus (Edgar et al., 2002) and are accessible through GEO Series accession number GSE152143 (https://www.ncbi.nlm.nih.gov/geo/query/acc.cgi?acc=GSE152143). Of 91,978 total transcripts, 47,122 were annotated in the *S. bicolor* genome. Further functional annotation using known genes in the greater Poaceae was obtained to add context to differentially expressed genes between treatments. There were 278 differentially expressed genes between deficit low and control low, 158 of which were down-regulated. 422 genes were differentially expressed between deficit mid and control low, 298 of which were down-regulated. Finally, 684 genes were differentially expressed between deficit mid and deficit low treatments, 417 of which were down-regulated. Differential expression tables are available in supplemental information (Supplementary Tables 1–3). Of these many genes, only seven genes of interest were identified: XP_002445047.2 aquaporin NIP3-2, XP_021314143.1 beta-galactosidase 4 isoform X1, WAT1-related protein At2g40900, XP_021316558.1 salt stress-induced protein, XP_015614593.1 beta-amylase 3, XP_002453072.1 aquaporin PIP1-5, and XP_002452133.1 plasmodesmata callose-binding protein 2 (Figures 6A–G). The top ten GO enrichment terms are available for each treatment comparison in Supplementary Tables 4–6. Other than general cell housekeeping functions, Deficit Low had three differentially expressed “drug transmembrane transport” genes (GO:0006855) in contrast to Control Low. Deficit Mid again showed enrichment in “drug transmembrane transport” genes, as well as “cell redox homeostasis” (GO: 0045454), compared to Deficit Low. Deficit Mid samples showed significant overexpression of pathogen defense responses compared to Control Low (GO:0050832, GO:0042742, GO:0006952, GO:0006032, GO:0016998).

## Discussion

The clearest result of this study is that sorghum requires prior exposure to drought to generate root pressure. Other studies have found that pre-exposure to drought increased root pressure (Stiller et al., 2003; Barrios-Masias et al., 2015) but in this study, re-watered individuals in the control group did not produce significant root pressure (> 5 kPa, Figure 2). This suggests that the experience of drought is a required trigger for the mechanisms that generate root pressure, at least in sorghum, although root pressure may still be constitutive or diurnal after priming by drought.

Interestingly, beyond partial re-watering following drought exposure, the magnitude of root pressure did not continue to increase with more re-watering across all genotypes (Figure 3). The significant interaction between genotype and re-watering level indicates that there could be genotype-specific responses depending on water availability, but root pressure is not consistently related to re-watering quantity after drought across different genotypes. Thus, soil water content either does not drive root pressure by a linear or exponential function in sorghum as it did for *Zea mays* in Gleason et al. (2017), or the threshold for re-watering is substantially lower either for sorghum in general or specifically in response to the treatments applied in this study. The lower root pressure in two drought tolerant genotypes in response to 100% re-watering compared to 50% re-watering is particularly notable, suggesting that the generation of root pressure was inhibited, and worth further study.

Interestingly, very few biometric and no physiological traits were correlated with root pressure magnitude. Root pressure was too variable, even within genotypes, to be statistically explained by re-watering amounts or root traits. There may be genotype-specific positive relationships between fine root allocation and root pressure, but these remain largely qualitative (Figure 5). An exception was the genotype SC56, which had very small relative fine root investment but produced root pressure magnitudes equal to those of much larger plants with higher fine root allocation. Other studies have predicted that total bulk tissue quantity could contribute to maintenance of root, stem, or leaf pressure via tissue capacitance (Gleason et al., 2014; McCulloh et al., 2014). In this study, root pressure was significantly correlated with green shoot biomass, total shoot biomass, and total plant biomass but not root biomass, which is theoretically a major source of tissue water available to drive root pressure. The significant correlations of root pressure with total and aboveground green biomass, furthermore, suggests an agricultural yield benefit associated with increased root pressure under drought conditions (Figure 4, Supplementary Figure 5).

The specific adaptive function of root pressure in sorghum remains unclear. The magnitude of root pressures measured in the re-watered deficit treatment would be sufficient to pressurize a 6-m tall plant in the absence of transpiration, indicating that root pressure could refill daily embolisms in drought stressed plants (Comstock and Sperry, 2000). However, while photosynthetic rates measured prior to re-watering were correlated with leaf xylem embolism, the magnitude of root pressure did not correlate with either photosynthesis or the number of embolisms. Given the low variation in embolism counts (between 0 and 2 embolisms per leaf cross section) and the lack of difference between number of embolisms in the control and deficit treatments, it is possible that leaf xylem embolism is not a major issue for *S. bicolor*. It is also possible that deficit plants experienced greater numbers of embolisms prior to re-watering, and that 1 h at 50% ET constituted near-full refilling and recovery. It remains an important question whether root pressure is involved in embolism recovery in sorghum.

Exploratory transcriptomic analyses suggested a set of differentially-expressed genes between our three root pressure magnitude/watering treatments that could be involved in the creation of root pressure. The vast majority of these transcripts are of unknown function or code for cell housekeeping functions, pathogen defense, and synthesis of secondary metabolites. While drought is known to regulate expression of large and diverse suites of genes (Cohen et al., 2010; Kakumanu et al., 2012; Johnson et al., 2014), only a small set of genes in our differentially expressed subset had possible relevance to the creation and maintenance of root pressure. We found seven genes of interest whose expression involved significant up- or down-regulation in the Deficit Mid treatment.

### Aquaporins

Two aquaporins were differentially expressed between the three treatments—PIP1;5 and NIP2;3. While PIPs are known efficient water transporting channels, NIPs tend to move other substrates, such as small organic solutes and minerals (Martre et al., 2002; Maurel et al., 2015). In this study, PIP1;5 is strongly down-regulated in the deficit treatments. Similarly, Liu et al. (2014) observed that while water uptake, hydraulic conductivity, root pressure, and most aquaporin expression increase in concert in *S. bicolor*, PIP1;5 does not. In *Hordeum vulgare*, aquaporin expression was up-regulated as root hydraulic conductivity was down-regulated, indicating that gene expression of aquaporins (as compared to gating or remobilization of aquaporins) may not always drive root hydraulic conductivity (Saini and Fricke, 2020). It is also possible that the down-regulation of PIP1;5 prevents flow of water back into the soil, supporting the buildup of root pressure, as opposed to facilitating water uptake from the soil. The second differentially expressed aquaporin, NIP2;3, is not clearly involved in root pressure generation: again, it is down-regulated in the Deficit Mid treatment (Figure 6F), and is known to transport arsenic, but not water, in rice and octopus (Chen et al., 2017). It should be noted that phenotypes resulting from differential aquaporin gene expression can be inconsistent between varieties of one species, and very often between species (Alexandersson et al., 2005; Grondin et al., 2016; Kadam et al., 2017).

### Membrane Transport Proteins

The WAT1-related protein, a plasma membrane transporter also known as UmamiT11 in *A. thaliana*, is localized in the loading/unloading domains of the vasculature in direct contact with the protoxylem, companion cells, and sieve elements (Müller et al., 2015). Previous work on this protein has focused on its role in endosperm growth and amino acid transport, although WAT1 is an auxin transporter. It is up-regulated in the Deficit Mid treatment relative to the Low root pressure treatments and may play a role in setting up an osmotic gradient in the vascular bundles and parenchyma to draw in water from the soil (Figure 6C). Similarly, the salt stress-induced protein (saLT) is a plasma membrane transporter with a strong increase in expression in salt-stressed *O. sativa* and *A. thaliana*, and here is up-regulated in the Deficit Mid treatment (Figure 6B). It is a lectin that likely binds carbohydrates and plays a role in the regulation of cell osmotic potential (UniprotKB- Q0JMY8, Garcia et al., 1998).

### Carbohydrate Metabolism

Carbohydrate metabolic enzymes strongly affect the osmotic potential of the cytosol and vacuole (Cram, 1976; Smeekens and Rook, 1997). We observed two significantly differentially expressed enzymes with opposite expression patterns—beta-amylase 3 and beta-galactosidase 4 (Figures 6G,D). Beta-amylase hydrolyzes starch to di- and monosaccharides, while beta-galactosidase catalyzes the hydrolysis of glycoproteins into monosaccharides. It is not clear why starch hydrolysis would cease while glycoprotein hydrolysis increases in plants with higher root pressure, but another study found that the gene expression of these two enzymes move counter to one another in the context of cold de-acclimation (Oono et al., 2006). It is possible that these enzymes are located in different cell types within our homogenized fine root samples and are somehow working additively to structure an osmotic potential gradient from soil to xylem.

### Vascular Transport

The plasmodesmata callose-binding protein 2 (PCDB) is significantly up-regulated in the deficit treatments relative to the control (Figure 6A). In other studies, PCDB1 was observed to occlude plasmodesmata (the symplastic connections between plant cells) when up-regulated, while PCDB2 mutants did not, although both proteins are localized in the plasmodesmata and bind to callose *in vitro* (Simpson et al., 2009). While it is tempting to associate higher root pressure with increased symplastic flow, closure of plasmodesmata may play an important role in preventing leakage of water down the osmotic gradient and back into drying soil and may be required for the creation of root pressure (Schenk et al., 2021).

## Future Directions

It will be critical to follow up on this study with diverse tools for investigating mechanisms involved with the recreation of root pressure. A larger, carefully replicated transcriptomic sampling of the same *S. bicolor* genotypes, or a subset therein would be valuable. Given the potential variability of mechanisms and transcriptomes between individuals, cultivars, and species in this and other studies, it may be difficult to rigorously pinpoint one set of mechanisms or genes that clearly drives, or responds to, root pressure generation. Moreover, as we have seen in many works studying evolutionary trade-offs in plant hydraulic traits, a wide variety of traits and phenologies can be variously adopted by different genotypes to achieve the same result, be it avoidance or tolerance of drought, or maintenance of an embolism-free hydraulic network (Watkins et al., 2010; Ocheltree et al., 2015; Han et al., 2017). The mechanisms and specific genes that regulate root pressure in *S. bicolor* may not bear resemblance to those that regulate root pressure in other species, such as *Zea mays*, although the creation of root pressure may achieve the same result of improved performance during drought.

## Data Availability Statement

The datasets presented in this study can be found in online repositories. The names of the repository/repositories and accession number(s) can be found below: https://www.ncbi.nlm.nih.gov/geo/, GSE152143;https://www.ncbi.nlm.nih.gov/geo/, GSM4604668; https://www.ncbi.nlm.nih.gov/geo/, GSM4604669; https://www.ncbi.nlm.nih.gov/geo/, GSM4604670; https://www.ncbi.nlm.nih.gov/geo/, GSM4604671; https://www.ncbi.nlm.nih.gov/geo/, GSM4604672; and https://www.ncbi.nlm.nih.gov/geo/, GSM4604673.

## Author Contributions

SD designed the experiments, collected data, analyzed data, and wrote the manuscript. LC designed the experiment, collected data, and edited the manuscript. NF designed the experiment and collected data. RB, JW, TP, JB, CJ, and SG collected data. JIC analyzed data. All authors contributed to the article and approved the submitted version.

## Conflict of Interest

The authors declare that the research was conducted in the absence of any commercial or financial relationships that could be construed as a potential conflict of interest.
