# Supplementary figures and images for: Drought-Induced Root Pressure in Sorghum bicolor
Source: Front Plant Sci. 2021 Feb 3;12:571072. doi: 10.3389/fpls.2021.571072 (PMC7886691; doi:10.3389/fpls.2021.571072)

Schematic of experimental design in the greenhouse

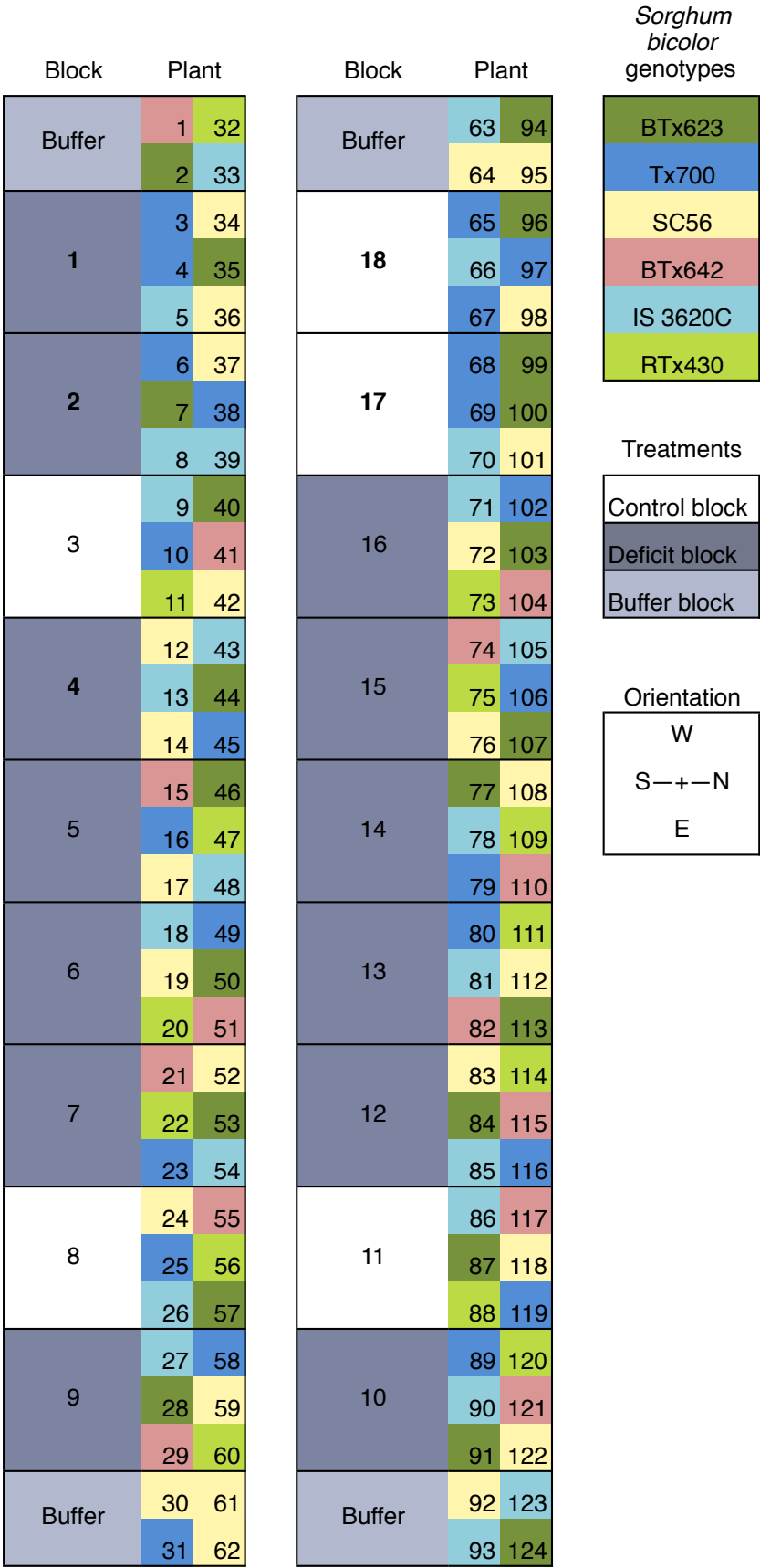

Supplement: Supplementary file 7 [file Image_1.pdf]

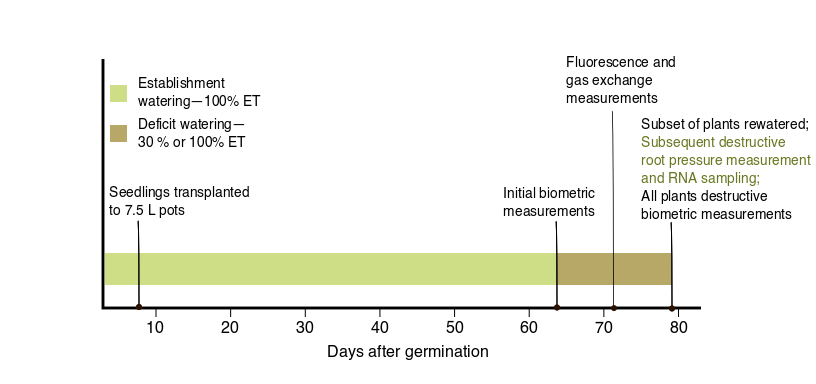

Supplement: Supplementary file 8 [file Image_2.png]

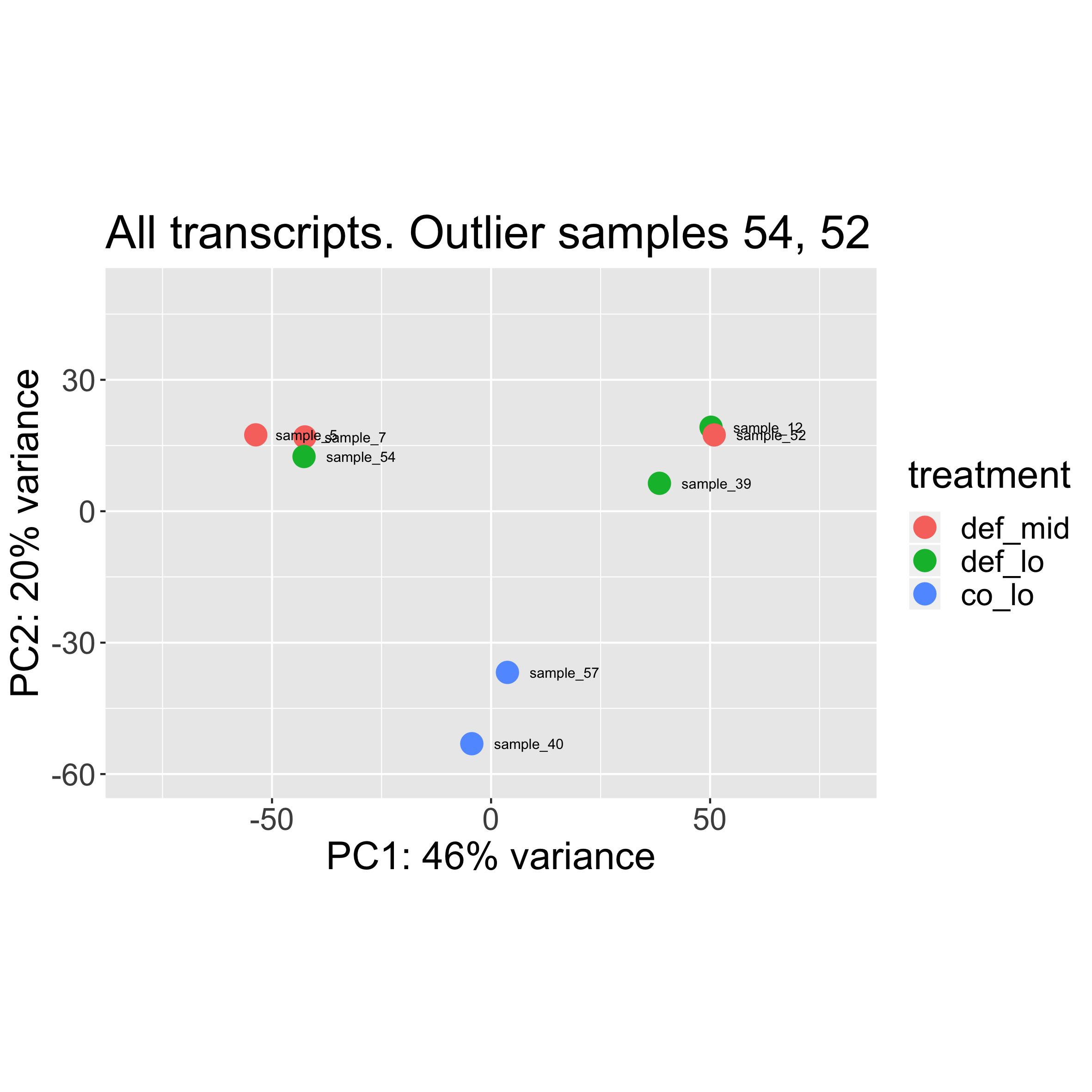

Supplement: Supplementary file 10 [file Image_4.png]

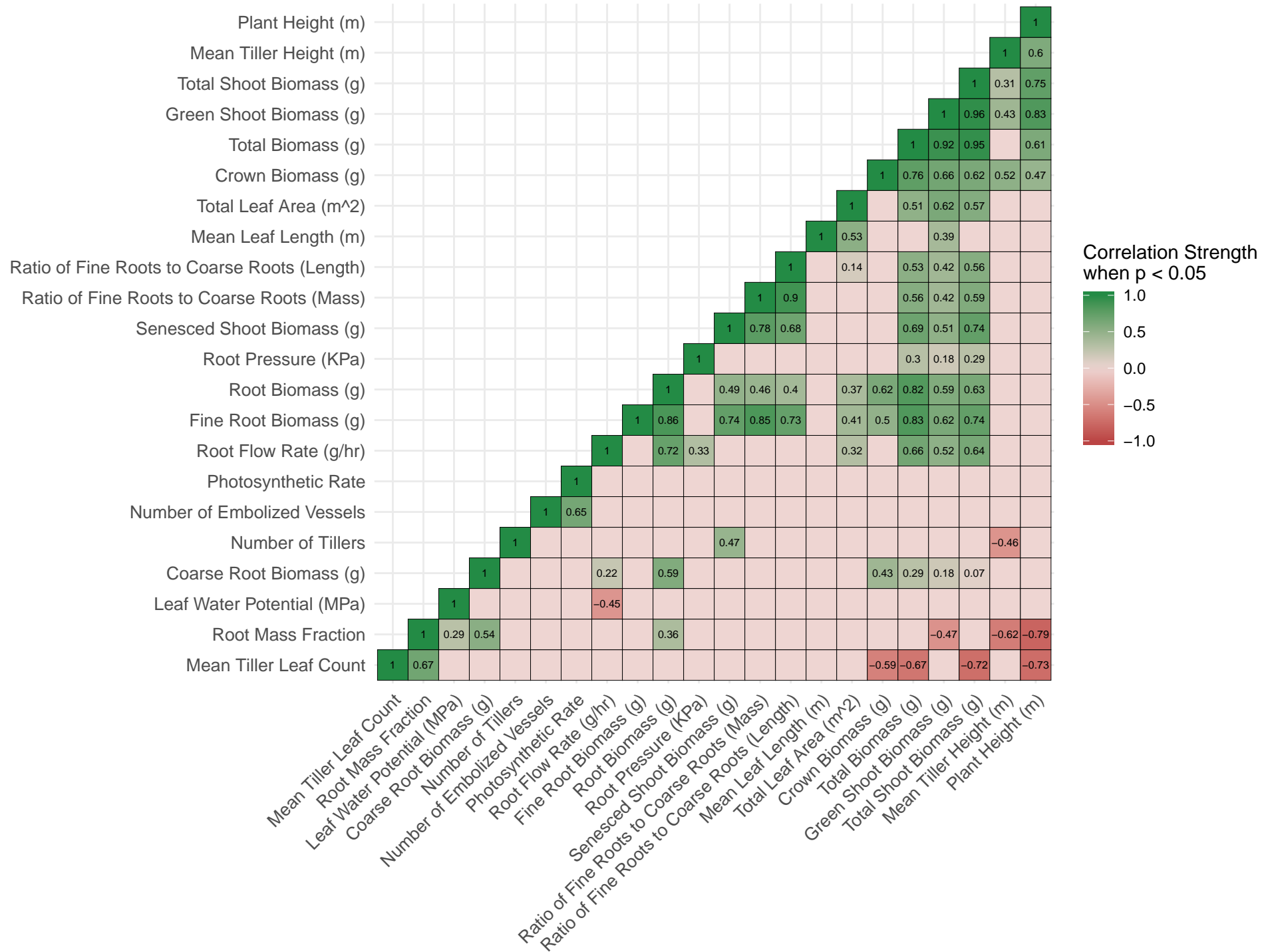

Supplement: Supplementary file 11 [file Image_5.pdf]
