# Supplementary material for: Drought-Induced Root Pressure in Sorghum bicolor
Source: Front Plant Sci. 2021 Feb 3;12:571072. doi: 10.3389/fpls.2021.571072 (PMC7886691; doi:10.3389/fpls.2021.571072)
Supplement: Supplementary file 9 [file Image_3.pdf]

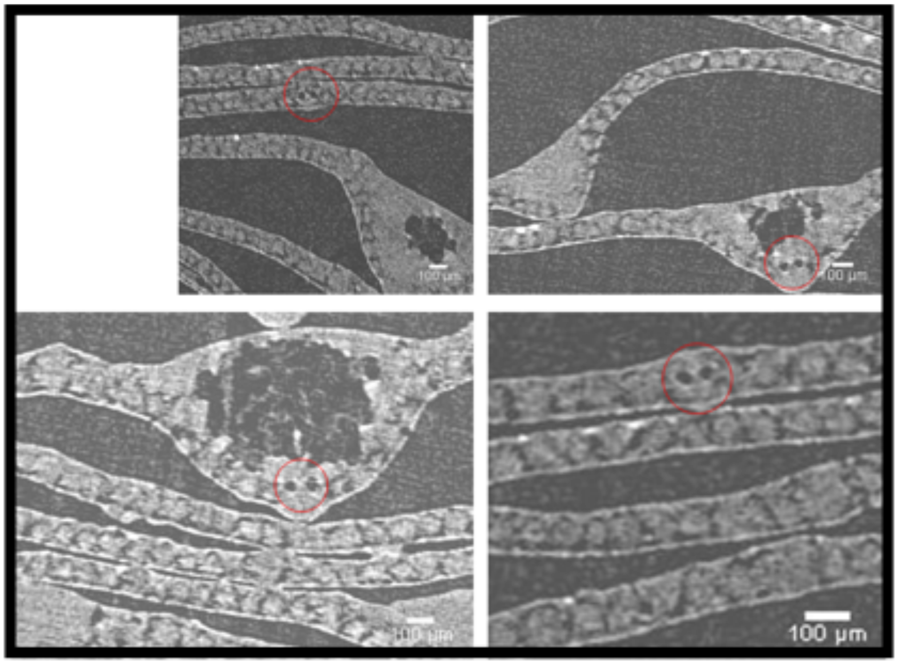

Figure S3. Microphotograph from µCT scan showing cross section of multiple ***S. bicolor*** leaves with central midribs.
